# A Rare Case of Signet Ring Cell Colon Cancer Presenting as Adult Colorectal Intussusception

**DOI:** 10.1155/2022/5271611

**Published:** 2022-02-08

**Authors:** D. A. Gaskin, A. Reid, M. O'Shea, P. S. Gaskin

**Affiliations:** ^1^Queen Elizabeth Hospital, Barbados; ^2^Faculty of Medical Sciences, University of the West Indies, Cave Hill, Barbados

## Abstract

Signet ring cell carcinoma of the rectum is rare and typically presents with advanced disease. We report a case of a 68-year-old man who presented with left lower quadrant pain and was found to have signet ring cell carcinoma with intussusception. This case is unusual because of its polypoid growth pattern and apparent early pathological stage. We discuss the differential diagnoses and prognosis.

## 1. Introduction

Signet ring cell carcinoma of the colon and rectum is rare with a reported incidence of 0.8 to 2.6% [1]. The tumour is characterized by abundant intracellular mucin with peripherally displaced nuclei. These cells must account for greater than 50% of the lesion for a tumour to be classified as signet ring cell carcinoma [2]. Signet ring cell carcinomas are reported to be more aggressive than other histological subtypes of colorectal carcinoma and are usually detected at a more advanced stage. The tumour typically has an endophytic/infiltrative growth pattern which results in a delayed presentation. Signet ring cell carcinoma with an exophytic/polypoid growth pattern, although uncommon, may present at an earlier stage [3]. The occurrence of this tumour is associated with a 14% incidence of inflammatory bowel disease, but family history is not strongly associated. Most cases are sporadic and occur at a younger age than typical colorectal adenocarcinoma [4].

## 2. Case Presentation

**Figure 1:**
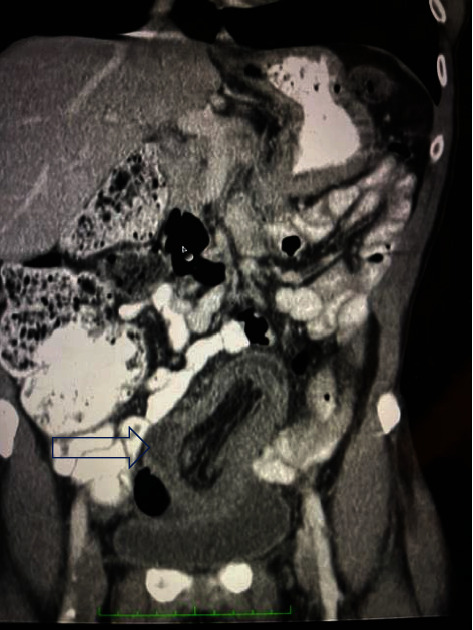
Coronal view of abdominal CT scan showing colorectal intussusception.

**Figure 2:**
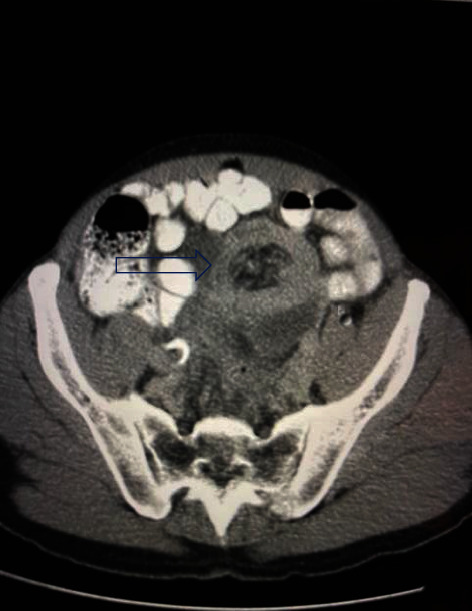
Axial view of the abdominal CT scan showing intussusception.

We present the case of a 68-year-old male admitted to the Accident and Emergency department with intermittent periumbilical pain for two days. There were no symptoms of intestinal obstruction. On examination, the patient was haemodynamically stable with tenderness in the left iliac fossa. There was no evidence of abdominal distension and bowel sounds were present. A diagnosis of diverticulitis was postulated. The patient was referred for an abdominal ultrasound which showed a left iliac fossa bowel mass and an abdominal CT scan was recommended. The CT scan revealed a “bowel within bowel” configuration in the pelvis involving the sigmoid colon into the rectum with fluid and air trapped within. Sigmoid wall thickening with pericolonic fat stranding was noted (Figures 1 and 2). The findings were suggestive of a colorectal intussusception. The lead point of the intussusception was not seen. In addition to the aforementioned findings, a small lytic lesion in the left iliac bone was seen and a bone scan was recommended. Colonoscopy showed a mass at 20 cm which was biopsied. Histological evaluation of the biopsy was in keeping with a mucinous adenocarcinoma.

**Figure 3:**
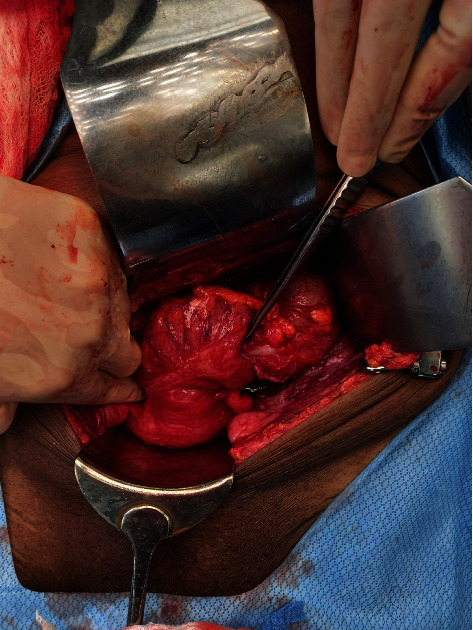
Intraoperative view of the colorectal intussusception.

**Figure 4:**
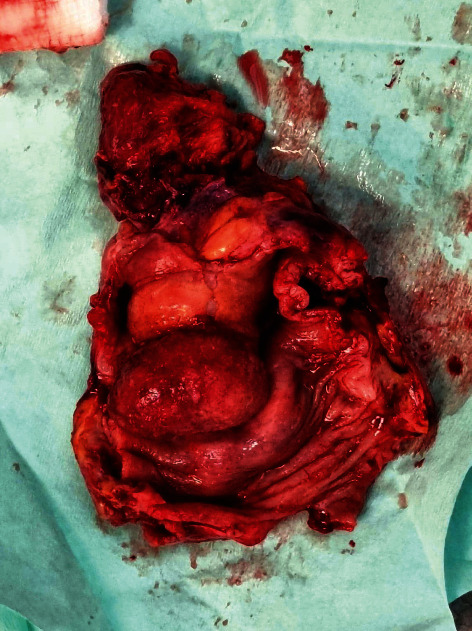
Gross specimen of the polypoid signet ring carcinoma.

The patient was referred for a surgical consult. The general examination was normal, the abdomen was soft and nontender, no masses were palpated and the digital rectal examination was normal. The patient was assessed as having a sigmoid colonic intussusception into the rectum with a colonic carcinoma as a lead point. The CT scan of the chest revealed no metastases. The bone scan, urea, electrolytes, liver function tests and complete blood count were within normal limits. The carcinoembryonic antigen level was 13.20 ng/ml (normal range: 0.00–5.00 ng/ml). The patient consented to an anterior resection. At surgery through a lower midline incision, the intussusception was noted (Figure 3). There were dense adhesions between the rectum and the pelvis side wall. The anterior resection was performed without reduction of the bowel as there was a pathological lead point (Figure 4). The anastomosis was performed using a handsewn technique with interrupted 3/0 vicryl. An appendicectomy was performed as the vermiform appendix appeared inflamed. The postoperative period was uneventful. The patient was referred to oncology for continued care.

## 3. Pathology

**Figure 5:**
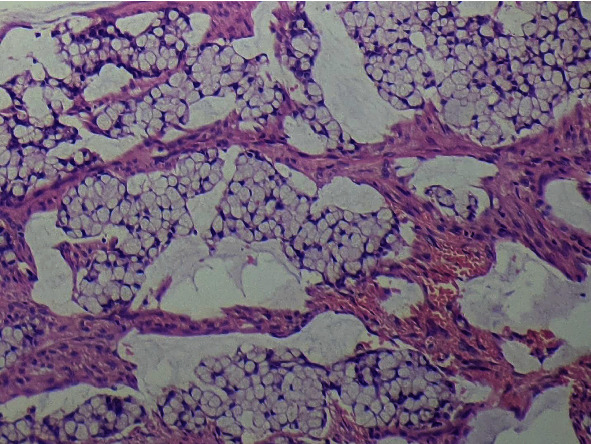
A photomicrograph of primary signet ring cell carcinoma of the sigmoid colon (HE; ×100).

The initial biopsy of the tumour was reported as a mucinous carcinoma. The histology of the anterior resection specimen revealed a pT3 N0 Mx signet ring cell carcinoma (Figure 5). An exhaustive search of the pericolic adipose tissue revealed four lymph nodes all of which were negative for metastases. There was no evidence of lymphovascular or perineural invasion. All resection margins were free of tumour. Immunohistochemistry (IHC) revealed proficiency for all MMR proteins. Molecular testing revealed the absence of KRAS mutation in codons 12, 13, 59, 61, 117, and 146. The appendix was normal.

## 4. Discussion

Adult onset intussusception accounts for 5% of all intussusceptions and 1% of cases of bowel obstruction. Most adult cases are colocolonic and up to 60% of these are caused by primary colonic adenocarcinoma [5, 6]. Signet ring cell colon carcinoma, like its more common gastric counterpart, usually presents as a diffuse infiltration of the bowel and was originally described by Laufman and Saphir as a linitus-type carcinoma of the colon [7]. Intussusception is an unusual clinical presentation of this tumour. In the published literature, we were only able to find three cases, all of which occurred in adult patients < 50 years [8–10].

Signet ring cell carcinoma of the colon is broadly defined by the presence of >50% signet ring cells. The entity can be further subdivided into mucin-poor and mucin-rich variants based on the percentage of extracellular mucin. The mucin-rich variant has >50% extracellular mucin and is associated with nonsignet ring areas of mucinous carcinoma. This variant also presents at a lower stage and has fewer adverse prognostic factors like perineural and lymph-vascular invasion. The index case may be considered mucin rich as collections of signet ring cells float in extracellular mucin pools. There were also focal nonsignet ring areas of mucinous carcinoma [11].

Signet ring cell carcinoma is related to mucinous adenocarcinoma of the colon but has distinct molecular features. Both mucinous adenocarcinoma and signet ring cell carcinoma of the colon are associated with microsatellite instability and have a lower frequency of KRAS mutations than the conventional primary colonic carcinoma [12]. However, signet ring cell carcinoma of the colon diverges from mucinous adenocarcinoma with the acquisition of genomic alterations that facilitate a more invasive phenotype. These include loss of expression of CDH1 (cadherin-1) and upregulation of genes resulting in epithelial-mesenchymal transition. CDH1 encodes the protein E-cadherin (epithelial cadherin) which is important in maintaining cell-cell adhesion and regulating cell proliferation via the WNT pathway [13, 14].

The differential diagnostic categories to consider in a colon carcinoma with signet ring morphology include primary mucinous adenocarcinoma, metastatic signet ring cell carcinoma and a benign lesion with signet ring cell change. Primary mucinous adenocarcinoma is a distinct subtype of colonic carcinoma that is characterized by the presence of abundant extracellular mucin comprising >50% of the tumour volume [15] Primary mucinous adenocarcinoma can have signet ring cells and therefore mimic a signet ring cell carcinoma in a small or limited biopsy. Both tumours can also have abundant extracellular mucin [11, 15]. Hence, the distinction of mucinous adenocarcinoma from signet ring cell carcinoma of the colon is based on the percentage of signet ring cells present. A colon carcinoma with >50% signet ring cells is classified as a signet ring cell carcinoma regardless of the volume of the extracellular mucin. This distinction is important as signet ring cell carcinoma is considered a distinct entity with a worse prognosis than mucinous adenocarcinoma even when there is a minor component of signet ring cells in the latter [2]. The abundant extracellular mucin in this case of signet ring cell carcinoma made differentiation from mucinous adenocarcinoma difficult on the initial biopsy. However, the presence of >50% signet ring cells in the resection specimen clinched the diagnosis.

Morphologically, a mucin-poor variant of signet ring cell carcinoma can mimic a metastatic signet ring cell carcinoma. Both tumours can have poorly cohesive cells with a diffusely infiltrative pattern. The metastatic tumour will have a different immunohistochemical profile. The coexpression of CK20, CDX2, and SATB2 will favour a primary colonic adenocarcinoma. However, this must be correlated with clinical and radiological findings as some of the antigens are also expressed by other gastrointestinal carcinomas [16, 17]. Diffuse gastric carcinoma is the most common metastatic signet ring cell carcinoma which can be distinguished by the presence of less extracellular mucin and a more diffuse pattern of growth than a significant proportion of primary signet ring cell carcinoma. The transverse colon is the favoured metastatic site for diffuse gastric carcinoma.

Nonneoplastic colonic lesions can also show signet ring cell change that mimics signet ring cell carcinoma. Most published cases are associated with pseudomembranous colitis [18, 19]. Absence of overt nuclear atypia and transperitoneal dissemination will often distinguish signet ring cell change from signet ring cell carcinoma. However, this can be a challenge in small biopsies or early-stage signet ring cell carcinomas which can be compounded by cytokeratin expression in nonneoplastic signet ring cells. The exact mechanism of this change in pseudomembranous colitis is uncertain. However, loss of expression of E-cadherin, abnormal expression of p53 and a high ki67 proliferative index can be used to differentiate signet ring cell carcinoma from signet ring cell change in challenging cases [18, 19].

The aggressive biology of these tumours explains their advanced stage at presentation. However, there have been 26 published cases that presented at an early stage with limited locoregional extension and no distant metastases. In three of these cases, an adenomatous component was seen providing supporting morphological evidence for an adenoma-signet ring cell carcinoma sequence [20]. Of note, 10 of the 26 published cases in this review had a polypoid or exophytic pattern of growth like the index case. This corroborates with other published series of signet ring cell carcinoma in which exophytic signet ring cell carcinomas with a polypoid growth pattern presented at an earlier stage [3]. Exophytic lesions are more likely to obstruct the lumen of the bowel and may therefore present earlier than diffusely infiltratively lesions especially at distal sites. The existence of precursor and early lesions of primary signet ring carcinoma suggests that a colon cancer screening program can potentially prevent mortality and morbidity in some of these cases.

Signet ring cell carcinoma remains an independent adverse prognostic risk factor even when correcting for tumour stage [21]. Hence, this patient is still considered a candidate for adjuvant therapy in spite of the pathological N0 status and lack of high-risk features like perineural and lymph vascular invasion. Pulmonary and hepatic metastases are uncommon in signet ring cell carcinoma unlike conventional adenocarcinoma. Metastases to the brain and bone including bone marrow are more common with these tumours [22]. Hence, reliance on traditional radiological imaging with MRI and CT may result in understaging, and occult malignancy or subclinical lesions may be missed. A more comprehensive approach that includes serum CEA levels along with bone scan or PET-CT should be performed to detect early residual and/or metastatic disease. There was no gross intraoperative evidence of peritoneal metastases, and a bone scan revealed no radiological evidence of bone metastases.

The late age of onset, polypoid growth pattern and lack of evidence of locoregional or distant spread are unusual features of signet ring cell carcinoma illustrated in this case. Histologically, this tumour exhibited abundant extracellular mucin. This feature is rare for a microsatellite-stable signet ring cell carcinoma and may influence the outcome of this tumour. This unique presentation adds to the diverse clinicopathological profile of colonic signet ring cell carcinoma in the literature and will hopefully aid other practitioners in the correct diagnosis and management of this clinically significant but poorly understood rare subtype of colon cancer.

## 5. Conclusion

This signet ring carcinoma is a rare tumour which presented as a mass as opposed to the typical infiltrative growth and appears to be at an early stage and limited to the bowel wall. This case report contributes to the current literature on this unusual and poorly understood subtype of colonic carcinoma. To the best of our knowledge, the index case is the oldest to have presented with a signet ring cell carcinoma and intussusception in current literature.

## Data Availability

The data used to support the findings of this study are included within the article.
